# What do older people experiencing loneliness think about primary care or community based interventions to reduce loneliness? A qualitative study in England

**DOI:** 10.1111/hsc.12438

**Published:** 2017-02-23

**Authors:** Kalpa Kharicha, Steve Iliffe, Jill Manthorpe, Carolyn A. Chew‐Graham, Mima Cattan, Claire Goodman, Maggie Kirby‐Barr, Janet H. Whitehouse, Kate Walters

**Affiliations:** ^1^ Primary Care and Population Health University College London London UK; ^2^ Department of Primary Care & Population Studies University College London London UK; ^3^ Kings College London London UK; ^4^ Primary Care & Health Sciences Keele University Staffs UK; ^5^ School of Health, Community and Education Studies Northumbria University Newcastle upon Tyne UK; ^6^ CRIPACC Faculty of Health and Human Sciences University of Hertfordshire Hatfield UK; ^7^ Lay Member London UK; ^8^ Lay Member Essex UK

**Keywords:** community based interventions, loneliness, older people, primary care

## Abstract

Loneliness in later life is a common problem with poor health outcomes. However, interventions to prevent or ameliorate loneliness have a weak evidence base. The views of older people experiencing or at risk of loneliness in the community are important in identifying features of potential support, but have been little studied. Twenty‐eight community dwelling people, aged 65 and over who reported being ‘lonely much of the time’ or identified as lonely from the de Jong Gierveld six‐item loneliness scale in a larger study, participated in in‐depth interviews, between June 2013 and May 2014. Views and experiences on seeking support from primary care and community based one‐to‐one and group based activities, including social and shared interest groups, were explored. Interviews were recorded and transcribed. Thematic analysis was conducted by a multidisciplinary team, including older people. Using two different measures of loneliness enabled a spectrum of loneliness experience to be explored. Two‐thirds of the participants were the ‘younger old’ and all were able to leave their homes independently. Older people with characteristics of loneliness were generally knowledgeable about local social and community resources but, for the majority, community and primary care based services for their loneliness were not considered desirable or helpful at this point in their lives. However, group based activities with a shared interest were thought preferable to one‐to‐one support (befriending) or groups with a social focus. Descriptions of support as being for loneliness and specific to older people discouraged engagement. Older people experiencing or at risk of loneliness did not consider that primary care has a role in alleviating loneliness because it is not an illness. They thought primary care practitioners lack understanding of non‐physical problems and that a good relationship was necessary to discuss sensitive issues like loneliness. For many, loneliness was a complex and private matter that they wished to manage without external support.

**What is known about this topic**

- Loneliness in later life is a common problem, with poor health outcomes and implications for public health.
- Interventions to prevent or ameliorate loneliness have a weak evidence base.
- We have a limited understanding of how older people experiencing loneliness view services aiming to reduce this concern.

**What this paper adds**

- Older people with characteristics of loneliness generally know about local resources but do not consider services they perceive as being for ‘lonely older people’, as desirable or helpful.
- Group‐based activities with a shared interest are preferred to one‐to‐one support or social groups.
- Older people experiencing or at risk of loneliness may not consider that primary care has a role in alleviating this.

## Background

Loneliness in later life is increasingly considered a public health problem (WHO 2002; DH 2012). It has a prevalence of 16%–35% in those aged 65 and over, rising to up to half of those over 80 years, with severe loneliness (lonely all or most of the time) occurring in between 5% and 13% of the older community dwelling population in the UK (Savikko *et al*. 2005, Victor *et al*. 2005, Luanaigh & Lawlor 2008, Age UK 2010).

Loneliness is a subjective experience; an emotional and unpleasant response to a lack of satisfactory companionship (Heinrich & Gullone 2006). In later life, loneliness is linked closely to other experiences associated with ageing, such as loss of family and friends and declining health and income, as well as more recent socio‐demographic trends such as longevity, living alone for longer, relationship breakdown, and changes to families and communities (Age UK Oxfordshire 2011, Bernard 2013, Nicolaisen & Thorsen 2014). The links between loneliness and its harmful physical and mental health consequences are widely reported (Stuck *et al*. 1999, Savikko *et al*. 2005, Victor *et al*. 2005, Iliffe *et al*. 2007, Luanaigh & Lawlor 2008) and include increased risk of mortality (Lyyra & Heikinnen 2006). Depression and loneliness in older people are strongly associated (Green *et al*. 1994, Cacioppo *et al*. 2006, Golden *et al*. 2009), while loneliness seems an independent risk factor for future depression (Heikkinen & Kauppinen 2004).

Despite these associations, the role of primary care in reducing loneliness has not been clearly delineated and there is little indication of what it can offer above identifying and treating associated depression. The relevance of loneliness to primary care is clearer. Loneliness has been independently associated with increased primary care consultations (Ellaway *et al*. 1999), emergency (but not planned) hospitalisation among community dwelling older adults (Molloy *et al*. 2010) and early moves to long‐term care (Russell *et al*. 1997, Savikko *et al*. 2010).

Given the frequency of consultation with primary care, social prescribing may be a way that primary care practitioners can refer patients to non‐clinical community based sources of support. Social prescribing aims to promote integration between health and social care services with the voluntary and community sector (DH 2006) and the range of community options available commonly includes activities aimed at those experiencing or at risk of loneliness, such as befriending schemes. However, evidence of the effectiveness of social prescribing is currently limited to evaluations of pilot projects and little evidence on cost‐effectiveness is available; a rapid appraisal found little evidence on social prescribing programmes to inform commissioning (Centre for Reviews and Dissemination 2015).

For several decades in the developed world, welfare state and voluntary sector groups have sought to alleviate loneliness among older people (Means & Smith 1999). Currently in the UK, three main types of community based services to alleviate loneliness are common. These may be (i) run by local government as part of social services or community resources; (ii) run by local government or other public sector funded voluntary sector organisations; or (iii) offered by self‐funding community, self‐help and voluntary bodies that receive no/little state support but are linked to neighbourhood, leisure, self‐help, educational, occupational or faith groups (Moriarty & Manthorpe 2012). The activities undertaken may be individually or group focused, with one‐to‐one home‐based befriending being at one end of this spectrum and large‐scale social or educational groups at the other.

There has been very little high‐quality research into the effectiveness of community based interventions specifically designed to reduce loneliness and social isolation in later life (Findlay 2003, Cattan *et al*. 2005, Frost *et al*. 2010, Dickens *et al*. 2011, Masi *et al*. 2011). A systematic review of the effectiveness of health promotion interventions for loneliness and social isolation among older people found that 9 of the 10 potentially effective interventions were group activities with educational or support input, and those that targeted specific groups were more effective, and that six of the eight ineffective interventions provided one‐to‐one support, advice and information, or health needs assessment (Cattan *et al*. 2005). More recent systematic reviews of the characteristics of effective interventions for social isolation (Dickens *et al*. 2011) and loneliness (Hagan *et al*. 2014) in older people have similarly reported that group based formats were more effective. In addition, interventions developed with a theoretical basis, groups offering social activity and/or support, and those in which older people are active participants were effective for social isolation (Dickens *et al*. 2011), and those involving new technologies, effective for loneliness (Hagan *et al*. 2014). A meta‐analysis to assess the strength of evidence of interventions to reduce loneliness found pre‐post and non‐randomised comparison studies yielded larger mean effect sizes compared to randomised comparison studies and in studies that used the latter design, the most successful interventions addressed maladaptive social cognition (Masi *et al*. 2011).

These systematic reviews are limited to quantitative outcome studies. Despite the range of services and activities with the remit of alleviating loneliness, the prevalence of loneliness in community dwelling older people has remained fairly constant over the last few decades (Victor *et al*. 2002, Honigh‐de Vlaming *et al*. 2014). With the limited evidence base for interventions, it is important that services take into account the views of older people experiencing loneliness. Most views on such interventions are from those already engaged with services (e.g. Cattan *et al*. 2003, Lester *et al*. 2012, Silver Line, 2015), or report views that are not service specific (Johnson *et al*. 2007). We therefore know little about what older people with loneliness in the general population actually want, if anything, from services to address loneliness.

This paper describes findings from a study that aimed to explore the perspectives of community dwelling lonely older people about seeking support for loneliness from primary and community based services and the features of these services which informed their views.

## Method

### Population and setting

This study was nested within the Well‐being Interventions for Social and Health (WISH) study (Medical Research Council funded) which explored the feasibility of embedding a health and well‐being risk appraisal system into primary care. The 454 participants were community dwelling older people aged 65 and over, registered and recruited from five English NHS primary care practices (three in a London Borough, two in a semi‐rural County). Participants completed a multidimensional, comprehensive self‐assessment postal questionnaire, including two loneliness measures. Participants were excluded if they lived in a long‐term care facility (care home), had a severe incapacitating, life‐threatening or terminal illness, were unable to provide informed consent or if an assessment would be considered burdensome.

### Sampling

Following the main study, all participants who identified as lonely at baseline or 6 months follow‐up, either because of their answer to the single stem question ‘Do you feel lonely much of the time?’ or because they scored two or above in the six‐item de Jong Gierveld Loneliness scale (de Jong Gierveld & van Tilburg 2006), were sent a postal invitation to interview. Unlike the single stem question, the statements in the de Jong Gierveld Loneliness scale do not include the word ‘lonely’. Interview recruitment continued until the main emerging themes were reinforced and to oversample for diversity in age, gender, socioeconomic status, ethnicity and severity of loneliness among participants.

### Data collection

An interview topic guide addressing the research questions was developed iteratively using knowledge of the literature and in consultation with the voluntary sector and older people representatives on the study team. Topics included experiences of loneliness, attempts to ameliorate loneliness made by themselves or others, including prompts on views of both one‐to‐one and group based support (social and hobby‐based/educational) if these did not arise naturally in the discussion, barriers and facilitating factors to reduce loneliness, the perceived role of professionals (including primary care) in reducing loneliness, and potential components of interventions designed to reduce loneliness in older people. Data reported in this present paper draw mainly from the responses to questions about primary care and community based services.

Participants were offered interviews in their home, the university or a local community venue of their choice. Interviews were audio‐recorded and transcribed verbatim with consent.

### Analysis

A thematic analysis was undertaken to identify key emergent themes and their meaning. Transcripts were read independently by nine members of the research team including lay members and analysed using a constant comparative approach including searches for disconfirming evidence (Spencer *et al*. 2014). Transcripts were read thoroughly to ensure familiarity with the data, and significant sections of text were identified, annotated and summarised to describe emerging themes, both *a priori* themes from the topic guide and those emerging from participants’ accounts. The themes were organised into higher and lower level themes in a thematic framework, discussed within the study team and the framework further refined. The clusters of themes were then referred back to the original transcripts for validation (Spencer *et al*. 2014). The overall interpretation of meaning and explanations were then developed and their implications considered, with input from the entire research team. NVivo 10 software was used to facilitate data management.

NHS Research Ethics Committee approval for the loneliness interviews was given by NRES Committee South East Coast ‐ Surrey.

## Findings

Twenty‐eight interviews were completed, lasting between 75 and 135 minutes. Nine participants (32%) reported being lonely much of the time and 19 (68%) were lonely based on their responses to the de Jong Gierveld six‐item scale alone. Almost half the sample lived with others. In addition to the socio‐demographic details presented in Table 1, it is noteworthy that all participants were able to leave their homes, albeit with some difficulty for a few.

**Table 1:** Socio‐demographic characteristics of sample and self‐rated loneliness (*n* = 28)

Characteristic	*n* (%)
Gender	
Female	18 (64)
Male	10 (36)
Age	
65–74	19 (68)
75–84	5 (18)
85+	4 (14)
Ethnicity	
White UK	25 (90)
Other	3 (10)
Living arrangements	
Lives alone	15 (54)
Lives with others	13 (46)
Lonely much of the time	
Yes	9 (32)
No	19 (68)
Lonely on de Jong‐Gierveld six‐item scale (two or above)	
Yes	27 (96)
Total	28 (100)

This sample of community dwelling older people who either self‐identified or scored as lonely on a validated scale was able to describe their understanding and experience of loneliness in detail. The overarching view expressed was that support from community and primary care based services for their loneliness was not something they desired or considered helpful, at this point in their lives.

The level of current or previous engagement with services and support was variable. Participants who had not sought support were able to share their considerations and perceptions of local resources or described their knowledge of such resources. Those who had previously or were currently engaged in social activities described the factors that facilitated or were a barrier to their involvement. Across the spectrum of community and primary care based services and activities, the extent to which their focus was explicitly on supporting loneliness, also varied. Within this sample of lonely older people, most had previous or ongoing involvement in shared interest or hobby‐based groups, that is, activities without an explicit focus on loneliness. Behind such general impressions lay other understandings; themes emerging from participant narratives are presented below by type of service/resources and are illustrated with quotes.

### Could befriending be for me?

Many participants were unaware of one‐to‐one befriending schemes that were running in their neighbourhood at the time of interviews, either face‐to‐face or by telephone, led by local voluntary sector groups. A few asked for details about such services, however others expressed uncertainties around the motivation, personality and compatibility of the individual volunteer, the idea of a volunteer/stranger coming to your home and concerns about the content of such conversations. As one woman explained:

The one‐to‐one I'm not too sure about; it just depends, doesn't it? I presume people who do that are quite extrovert and jolly‐jolly, and have a chat with you. It could be that you really took to somebody and found them easy to chat with. I mean, yes, I think they're great ideas but I don't know. (Int 19: Female, 65–74 years, lives with others)

A volunteer? Well, I'd be embarrassed actually. (Int 2: Male, 65–74 years, lives alone)

Barriers to taking part in such schemes were identified including the stigma of being identified as lonely and the associated stereotypes of people who use services for loneliness or isolation, and not wishing to see themselves within this group. Several of the younger participants (65–74 years) reported, ‘Not now, maybe later’ including those who reported being lonely much of the time:

I'm not that desperate yet! (Int 5: Male, 65–74 years, lives with others)

But what I'm saying is, ten years down the line, I might think that's a really good idea. At the moment, I'm saying it's not for me, but if I was isolated in this house and couldn't get out, yeah, I think that would be a lovely idea, but just not at the moment. I think I have to find my own way at the moment of doing things. (Int 3: Female, 65–74 years, lives alone)

None of the participants expressed a wish to access a telephone befriending service, stating either that they did not particularly like that type of communication or that they would just telephone someone they knew instead. Some described the usual ways in which they developed acquaintances in the local neighbourhood to indicate that they did not have a problem with social contact:

I feel something like that may grow from somebody I might meet, say when I go up to the market and so on, and then I'll see them the next week and say ‘Hello’ and then I'll see them the next week ‘How are you?’ and it may grow into something, but I don't see it being presented to me and my saying welcome. (Int 26: Female, 75–84 years, lives with others)

### ‘Social groups’ are for others

Themes related to social groups (groups convened for a primary social purpose) overlapped to some extent with views on befriending schemes. Purely social groups with little or no specific activity (e.g. lunch clubs, coffee mornings) were widely perceived as being for ‘lonely old people’ and most participants were reluctant to attend, or reported some negative initial experiences when they had previously tried them. Some considered they were in better physical health than those attending groups targeted for the ‘elderly’ or expressed a preference for the company of younger people. Two men who both lived alone, who described themselves as lonely much of the time and did not mention any meaningful relationships other than their children who lived at some distance, painted a picture of the type of the people they thought went to social groups and why they would not go themselves:

To tell you the truth, I'm not really interested in that. I don't want to sit down there and listen to Mrs Jones and her rheumatism, and old Fred Bloggs talking about his bleedin’ lumbago! (Int 7: Male, 65–74 years, lives alone)

Other participants reported similar negative views of such groups, for example as providing ‘tea and bingo’ for older people:

I just feel they're not for me. I feel that the level at which they work wouldn't satisfy me. You're painting a picture of people really who have gone into old age and accepted it and are not asking anything of life now, except to go and have a cup of tea with somebody in a little group. It wouldn't do. I'd be thinking what could I be doing at home? I'm not coming back here again, I'm sure. (Int 26: Female, 75–84 years, lives with others)

The very idea that a group would meet individual needs was questioned by some:

We're all very different and we've got different needs and so the support mechanisms have got to be completely flexible to take into account every individual, and they are all individual needs, aren't they really? (Int 16: Male, 65–74 years, lives with others)

As few participants had successfully engaged with groups or activities in which addressing potential or actual loneliness was explicitly part of their remit, limited information can be gleaned on what would facilitate further engagement with such activities. However, basic hospitality and being generous both in attitude and with refreshments were important, as described by one participant as a group member:

They're always coming round, ‘Would you like a biscuit? Would you like a sausage roll? Cakes?’ and there's always tea and coffee available … Yes, it is very good, very generous. (Int 23: Female, 85+ years, lives alone)

However, another participant who had volunteered (once and many years ago) said her contrasting experiences had subsequently deterred her from going along as a member:

I just didn't like the atmosphere at all … I think they were impatient and I think with very elderly people, you've got to be really patient. And I think maybe I saw the impatience of, ‘You've had two cups of tea already!’ I mean, whose business is that if she wants ten cups of tea! You know, and I just had the feeling, no, you know, it's not for me. (Int 3: Female, 65–74 years, lives alone)

### Having a common interest

Most participants had or were currently attending shared interest group activities and views on these groups contrasted to those described above. Having a shared interest (rather than meeting for purely social reasons) seemed to make it easier to become involved, as expressed by participants who regularly attended group‐based activities, such as exercise groups. These groups were valued for their expressed content and also the social element that developed. This suggests that people may be sociable, involved with others, indeed nearly half were also living with others, and also feel lonely. One participant in the study, who ran a popular exercise group for older people and who was clearly valued by her members, despite reporting that she herself felt lonely, shared some of the many techniques she employed:

That's why with some of my ladies, I've known them so well for so long, that when they become widowed, I just make sure I ring them up, send them cards, ‘We miss you. When are you coming back?’ you know? Because it would be so easy for them. I've got one at the moment who is just not coping very well at all. I ring her and say, ‘Come along, because I can have a laugh with you’. (Int 28: Female, 65–74 years, lives with others)

Group activities enjoyed in later life were often interests that had often been established earlier. Participants described features that facilitated or presented barriers to their current engagement. Already knowing or recognising others attending the group seemed to reduce feelings of social unease, even if members were not known people but just recognised. Perceptions of how a person is welcomed to groups, in particular on the first occasion and how they are run, for example, in a paternalistic manner, was also important. Two contrasting experiences suggested different reactions to joining a group:

The things that put me off them is that generally to the extent that I've seen them, what's going on in them (and people aren't even conscious of it) is a tiny bit of power‐play that in a group of people that have come together to do something, some people feel the need to ever so slightly take charge, and then have around them people who, just maybe in the way the thing is organised and run, if you join, you join on their terms. (Int 14: Male, 65–74 years, lives with others)

In the second example, one person who had been anxious about joining a new group described how she planned ahead to make the first visit easier:

I went on my own, because two people I know were on holiday, but I phoned the lady who runs it and she introduced me to some people. (Int 27: Female, 65–74 years, lives alone)

### What can primary care offer?

Overall, the appropriateness of discussing loneliness with primary care practitioners was questioned by participants. There was a strong view that loneliness is not an illness, and a perception that GPs lacked understanding of problems that were not physical health problems. A few exceptions to this were cited, for example, by people with co‐existing mental health problems such as depression and anxiety. This small minority who were more likely to consider talking to their GP about loneliness had good relationships with their general practice, were used to discussing their mental health problems and had generally received treatment. A smaller number had managed to develop a relationship with a member of the primary care team having lived in the area for a long time:

Well, for instance, coming up 2 years ago, my doctor put me down for a sort of refresher in CBT [cognitive behavioural therapy] … Yeah. I mean, he's very good; he's spent a lot of time with me. (Int 10: Male, 65–74 years, lives with others)

Many, however, felt that they did not have the close relationship with their GP that they thought necessary to talk about problems such as loneliness, although some had identified individual members of the practice team they could talk to or would consider doing so in the future:

Well, that would be the last place I'd want to go, you see; they're not very sympathetic. (Int 4: Female, 65–74 years, lives alone)

The practice nurse I was sort of seeing was very, very sympathetic to me; she was very, very nice and I talked to her about the things that were really bothering me and she was so sympathetic, but it was like really a one‐off. (Int 3: Female, 65–74 years, lives alone)

Participants were also aware of the constraints on GPs’ time:

There are many times when I would have liked to have had a discussion, but the appointments are just 10 minutes. (Int 25: Female, 65–74 years, lives with others)

Others felt that talking to the GP or nurse about emotional problems would be ‘wasting their time’ as other problems were considered more pressing or that the likely solutions offered would be pharmaceutical. For example, one participant who reported being depressed and very lonely said:

Well, really, there's nobody to talk to really, is there? You can't talk to your doctor about it, because they'll just turn around and say, ‘Here's a tablet!’ And I take enough of them now, and that's about all; there's nobody actually to talk to really. (Int 7: Male, 65–74 years, lives alone)

### Dealing with loneliness privately

Other themes emerged about the overall idea of involving ‘others’ in their loneliness. Situations or life events for which services or support for loneliness were deemed inappropriate by many included those in unsatisfactory relationships, those grieving the loss of a partner and/or those who had experienced worse episodes of loneliness at other times. For many, these feelings of loneliness, particularly for those grieving, were seen as a private matter and ones to be worked through alone. Some had considered bereavement counselling and tried it briefly but none of them had persisted with it or found it particularly beneficial. Others had a good understanding of their situation and were able to describe how they managed their feelings. For example, one participant described the stigma she would feel using services that supported older people who were lonely, and ultimately loneliness for her was a private matter that she would not consider talking to anyone about:

I know I've said, you know, I feel alone and isolated, but I'm not sure whether it would help me to talk about it. I think I know why I feel alone and isolated. I think I know, I don't need somebody to tell me if you like. (Int 3: Female, 65–74 years, lives alone)

## Discussion

### Summary

This is one of the first studies to explore perceptions and experiences of lonely older people on community based avenues of support, in which the sample had not been invited on the basis of their current use of services for loneliness or loneliness risk. Overall, participants held negative views about services and activities they perceived as being badged or targeted at ‘lonely older people’. Many had tried a range of activities and services and were able to report reasons why they had stopped engaging. In particular, reservations were expressed about befriending and purely social groups, with most expressing preferences for groups with an activity or purpose that is not primarily social, and ones that are not necessarily specific to older people. Primary care was not seen as a place to share feelings of loneliness, meaning that it is unlikely that all older people with loneliness will volunteer themselves or request ‘social prescribing’. For many, loneliness is a complex and private matter that they prefer to manage themselves.

### Comparisons with previous literature

Participants in this study were not engaging with services for loneliness such as befriending or many social groups and their views are likely to be different to the sizable number of people who do use these services (Windle *et al*. 2011). Lester *et al*.'s (2012) study of the views of older people engaged with befriending services reported characteristics of the service that people had found to be helpful were: good conversational skills and empathy in the befrienders, and opportunities for emotional support and reciprocal social exchange through safe, confiding relationships. These experiences address some of the concerns expressed by the sample in the current study about the type of people delivering the service and the service remit. Participants’ views on befriending schemes including an attitude of ‘not now, maybe later’ may also have been influenced by the fact that two‐thirds of participants in our study were ‘younger old’ (65–74 years), and all participants were able to leave their homes independently (although some were beginning to have difficulty in this), in comparison to the largely housebound and very old population engaged with befriending schemes (Lester *et al*. 2012).

Older people experiencing or at risk of loneliness did not consider that primary care has a role in alleviating this. Over and above the constraints of time and access, some participants were cautious about the possible medicalisation of and pharmaceutical response to loneliness. A good relationship was deemed necessary to discuss sensitive matters like loneliness, similar to the ‘active listening’ by healthcare providers proposed by Smith (2012) in her exploration of meaning and coping mechanisms for loneliness in community dwelling older adults. Johnson *et al*. (2007) explored the coping and prevention strategies for loneliness of individuals aged 50 and over recruited primarily from voluntary agencies. A significant number were currently/had previously received some form of health or social care services, but little was reported about the role of these services regarding loneliness and social isolation. The services were described as enabling living in a ‘physical sense’ rather than ‘enhancing the social experience’ (p. 44).

Although there is little comparable research in this area, these views resonate with the larger literature on the views of older people with depression seeking support from primary care. Older people are similarly reluctant to recognise and name ‘depression’ as a set of symptoms that warrants seeking support from primary care and they have limited expectations of treatment, which is assumed to be predominantly biomedical. This is partly due to perceptions of the role of the GP and also to previous negative experiences of help seeking (Burroughs *et al*. 2006, Chew‐Graham *et al*. 2012). From a primary care perspective, studies have reported that some GPs have mixed feelings about offering medication to address what they believed to be the consequences of loneliness and social isolation (Murray *et al*. 2006), namely depression, which is contrary to the expectations of many of the lonely older people in this study.

Many expressed views about the private nature of their feelings of loneliness and the desire to manage these without involvement of others. This resonates with the view that loneliness can be a range of feelings which people live with and experience and manage differently (Hauge & Kirkevold 2012) and challenges assumptions about being recipients of support in later life (Allen & Wiles 2014).

### Strengths and limitations of the study

Study participants were able to articulate a breadth of experiences of loneliness and their considerations in seeking support to help manage these negative emotions. One strength of this study is that it includes older people with different degrees or characteristics of loneliness, ranging from those who admitted being lonely to a researcher to those whose prior completion of a survey about health status in private had indicated that they were at risk of loneliness. Furthermore, most people had not engaged with services for loneliness and many said they had not spoken about their loneliness to anyone previously. The sample therefore included those with loneliness whose views may not previously have been heard.

In addition, the older people in the multidisciplinary research team contributed both personal and professional perspectives to the development of the topic guide and analysis and interpretation of the data, a further strength of this study.

In interpreting the views of participants in this study, it should be borne in mind that the sample was recruited from a larger study of health and well‐being in later life, and it may not represent the views of those who do not take part in such research. Two‐thirds of the sample were in the ‘younger old’ age group and all were able to leave their homes (with some difficulty in some instances), and so the research does not represent the views of those unable to leave their homes, and under‐represents the older, frailer population who are likely to express different views. There was a good spread of gender and socioeconomic status, but a smaller number of older people from black and minority ethnic groups, who may also hold different views.

### Implications for research

Further research should explore the views of older people with loneliness who are unable to leave their homes but are not in contact with services, in particular regarding one‐to‐one approaches such as telephone or face‐to‐face befriending, or use of the Internet. Loneliness was considered a mostly private matter, and we need to understand more about how older people can be supported to ‘self‐manage’ their loneliness. Research developing new interventions should consider the heterogeneity of views regarding services seen as being targeted for loneliness, and the need to take these into account in the design.

### Implications for policy and practice

Participants reflected a population whose needs are important to consider in the commissioning of services. Avoiding descriptions of services and activities as being for older people experiencing or at risk of loneliness may increase their accessibility and their acceptability. Features to emphasise that may encourage this group of older people to make community connections include: the ability to maintain interests established earlier in life, accessing groups with a shared interest which may allow a reciprocity, purpose and value to the exchange, geographical proximity to increase the likelihood of recognising others attending local groups, and other efforts to acknowledge and minimise the potential social unease particularly felt by some older people who may find groups difficult. This largely mobile and active group of older people with loneliness were mostly ambivalent about using befriending services, which supports targeting of these services on older people who are unable to leave their homes.

Our study also provides important evidence about isolation and loneliness, in that nearly half of the participants lived with other people and so would not necessarily be seen as socially isolated. Targeting social and other resources on older people living alone would likely miss this group. Older people were reluctant to seek help from their GP or practice nurse for loneliness, and social prescribing initiatives in primary care would require a pro‐active approach to identify people who may benefit.

## Conclusions

Older people with loneliness who are able to leave their homes appeared largely ambivalent about services with a primary social purpose, perceived as being targeted for ‘others’. More positive views were expressed of activity based groups. They perceived a very limited role for primary care, and for many their loneliness was a private matter that they wished to manage without external support.

## Conflict of interest

No conflicts of interest have been declared.
